# The British Juggernaut in India
*A Report on the Health of the First Bombay European Regiment (Fusiliers), from 1st of April, 1846, to 31st of March, 1854. By F. S. Arnott, M.D., Surgeon of the Regiment. "Transactions of the Medical and Physical Society of Bombay" for 1855.An Analysis of the later Medical Returns of European Troops serving in the Bengal Presidency from 1846 to 1854. By Hugh Macpherson, Assistant-Surgeon in the Governor-General's Body Guard. "Indian Annals of Medical Science". No. VIII. 1857.


**Published:** 1857-10

**Authors:** 


### SANITARY REVIEW.

OCTOBER 1857.

### THE BRITISH JUGGERNAUT IN INDIA.*

Some fifty years ago, a journey to the Indies in the East was looked on as one of those great adventures which belong only to men of unruly will and magnificent nerve. A kind of solemn mystery surrounded such an exploit: it was like a man with his eyes open travelling into the valley of the shadow of death. Mothers heard of their soldier sons being dispatched to India, and their hearts sank for ever. There was no return, no hope. The farewell was equivalent to death: it was too often a death, a living death, in so far as the ties of home and relationship were concerned. The idea was doubtless somewhat exaggerated, yet not over much. For, at periods more recent than the one we have specified, European regiments in India, especially those of the Company's service, have melted away like the spectres of a dream. A thousand strong men formed this year a regiment; a year passed, and one hundred and twenty-five new recruits were required to fill up the broken column; eight years passed, and not a man of the original thousand remained in that dissolving corps. It was the same regiment, in the same sense as the Irishman's knife—first a new blade, then a new haft, and so on interminably. Not much wonder, then, that the Indian field for distinction should be considered a great grave-ground; not much wonder that the recollection of such facts should still throw a gloom over Indian adventurers.

It was remarkable how long the main cause of this sudden decimation, and unscrupulous transformation of men into bungs and other Shakesperian analogues, lay hidden in the dirt

---

* A Report on the Health of the First Bombay European Regiment (Fusiliers), from 1st of April, 1846, to 31st of March, 1854. By F. S. Arnott, M.D., Surgeon of the Regiment. "Transactions of the Medical and Physical Society of Bombay" for 1855.

An Analysis of the later Medical Returns of European Troops serving in the Bengal Presidency from 1846 to 1854. By Hugh Macpherson, Assistant-Surgeon in the Governor-General's Body Guard. "Indian Annals of Medical Science". No. viii. 1857.

of ignorance and supineness. When men asked men why India was so fatal, the bugbear of an answer gushed out in two words—the climate. There wàs some truth in the saying, but more suppression of truth than truth itself. It was the climate, truly, in some measure; but it was the climate unstudied, and the man unprotected.

Had we not the authority of an author who has seen and has known, and who is not given to string the long bow and drive the long shaft, we should hesitate to describe what, in those past times, were the true influences and negligences which led to such an useless sacrifice of human life, or to show up the British Juggernaut in India. The Juggernaut was not personified in the warm climate, nor in the battle field, but in grand evils elsewhere—in evils social, in evils sanitary, in evils remediable.

Dr. Arnott's description of the European soldier in India in past days (twenty-five years ago), is too graphic and instructive to be passed over in silence.

"In earlier times the condition of the European soldier in India was in many respects deplorable. Severed, perhaps at an early age, and, it may be, on account of some youthful and venial error, from all who took an interest in, or could advise or direct him, and with all the happy recollections of his boyhood fresh in his mind, the finest feelings of his nature were doubtless soon chilled by intercourse with men who could little sympathise with him. With passions and bodily energies in full vigour; the idleness, the monotony, the restraint, and the necessary confinement of a barrack life, in an ungenial climate, were irksome in the extreme; whilst superior talents and mental faculties could only make him the more pine for joys irrecoverably forfeited. There were, then, few opportunites for mental improvement. Books were scarce, and newspapers, if procurable at all, were of old date, and little interesting. For a season or two after arrival, the earnestly inculcated injunctions of his friends, and his own faithful promises, to maintain a regular correspondence may have been religiously observed; and the interchange of letters may for a while have cherished all his former associations, and kept alive the desire to revisit the scenes of his youth. But without any opportunity of renewing those ties, it is no easy matter, even among the better educated, to continue this regularity of correspondence during twenty years of exile, and at the distance of half the circumference of the globe. Consequently, by the gradual loss of correspondents, by the tardiness and uncertainty of communication, and by various other accidents, letters became less frequent, and old familiar faces and things began to be less distinctly remembered. At length other interests, other views, and other acquaintances springing up, the old associations ceased to be recollected. In many cases, without doubt, the spirit became broken at the dim prospect of ever revisiting those early scenes, at the despair of meeting those early friends, and at the disseverance of all connection with home. From the real or apparent forgetfulness of relations, the youth may have become indifferent to character and self-respect. He may have endeavoured to drown the recollection of former error, of blighted prospects, of home, and of relatives, in drunkenness and debauchery, which too often terminated in an untimely death. In those times, moreover, everything connected with India was imperfectly understood by the people at home; and more especially by the classes from which recruits are usually obtained. Enlistment into the Honorable Company's army was then deemed equivalent to perpetual exile, or to a certain and early grave. It was too often the last desperate step of men of reckless character, or of ruined fortunes. Mothers, and other relations, consequently looked with dread upon the Indian service, and the better classes of recruits were reluctant to enter it. The prejudices against it unfortunately were not without foundation. The soldier's condition was not then, as now, an object of watchful solicitude. He was worse paid, worse fed, worse clothed, and worse quartered. His rights were ill defined, and little understood. He was more at the mercy of those in authority over him. Punishments were capriciously inflicted. They were often very severe, and not unfrequently of a degrading nature. Parades and drills were long and harassing, and in many of their minutiæ irksome and pedantic (as indeed they are to this day). The services of European soldiers were much required in the field; and regiments and detachments, afloat as well as on land, had to perform severe duty, with a tolerable quantity of hard fighting; whilst the men, ever brave, and every ready to meet and conquer their country's foes, rejoiced in the excitement of active service. On such occasions their gallantry may have been acknowleged, but that was not held as a reason for pampering them with indulgences at the end of the campaign. They who in the tented field had become inured to the fatigues, privations, and asperities incident to military life, could not always calculate on a period of rest, or on being sent to an eligible station; but, being deemed seasoned soldiers, were expected to surrender superior accommodation, climate, and quarters, in favour of others less thoroughly acclimated."

Such was the condition of the soldier in India when the decimation of Indian troops was so severe. The decimation was provided for; and a cruel series of experiments as to the forces that would kill, ended in supplying a world of scientific but dearly bought information.

Lord Ellenborough, in calling the attention of the House of Lords in 1853 to the decimation of Indian European troops, took occasion to compare favourably the troops of Her Majesty's service, with those in the service of the Company. His lordship shewed from the then existing returns, that, whereas a regiment belonging to Her Majesty required renewal each twelve years, a regiment belonging to the Honourable Company demanded such renewal, as we have before shewn, each eight years. Dr. Arnott, commenting on this statement, remarks that, at the time he wrote, 1854, such a contrast was probably not correct. In Her Majesty's Indian forces, so many improvements had then taken place, that, instead of 83⅓ recruits being required annually to keep up the strength of 1000, there were only needed 62½. In other words, a Royal regiment had to be renewed only once in sixteen, instead of, as formerly, once in twelve years. The same improvement had also, according to this writer, occurred, at least proportionately, in the Company's service; such, any way, was his impression.

It were fortunate for India, were these opinions of Dr. Arnott supported by other and later statistical evidence. Turning from his excellent record to the second paper named at the head of this article, we find in the medical returns of European troops serving in Bengal, for the eight years 1846-54, a series of deductions, which mournfully overcast the brightness of the preceding picture. Mr. Macpherson starts forth with a table, shewing the annual average loss from all causes, occurring amongst European troops in Bengal, for a period of eight years, *i. e.* from 1846 to 1854. And what is his summary? This: That the force serving in Bengal loses annually, from one cause or another, 9 per cent. of its numbers; a loss amounting to an annual average of 1,759 men out of 19,517, and to a gross total loss in the eight years of 14,072 men out of an army of 156,139.

Whatever, therefore, may be the position of affairs in the other Presidencies, it is clear that in Bengal a regiment of 1000 men dissolves away in about eleven years, even in favourable times; and this notwithstanding the improved conditions of the men in both services.

In relation to the improvements which have taken place in the Company's regiments, Dr. Arnott thus reports:—

"For many years improvement has been progressive, and the condition of the Honourable Company's European soldier has almost yearly undergone amelioration. His own never-failing gallantry in action, his devotion, his courage, and his patient endurance of toil and privation; and probably, also, the greater experience of his superiors—their better acquaintance with his wants and privileges, and their own increased responsibility, have secured him justice, and contributed in every way to benefit him. The soldier's duty is now more regular; he is less harassed upon parade, and he is not treated as an unreasoning, brutalised being; severity is not now considered essential to discipline; youthful delinquencies and indiscretions are not now visited by awards less calculated to restrain vice than to convert the thoughtless and unwary into hardened and irreclaimable offenders; and hence there is no motive for the discreditable, unfair, and unmilitary practice of concealing crime. At the same time, however, the smallest dereliction of duty or straightforward conduct, is at once checked by adequate punishments. Drill, duty, discipline, punishments, and the entire interior economy are reduced to a system, and do not depend upon the will or caprice of any one. The soldier is now better paid, better fed, better clothed, and better quartered (although in some of these particulars there is yet much room for improvement), and with rare (but occasionally very glaring) exceptions, he has now the regular tour of the different stations of the Presidency. Commensurate with the additions to his comforts, has been the improvement of the habits of the soldier in the Honourable Company's European army; which, combined with the reputation of its glorious achievements in war, and the high prizes it holds out, have attracted to its standard a better class of recruits; so that men of great ability and intelligence, of respectable birth, of superior social position, and of excellent education, now muster strong in the ranks of every regiment; whilst sobriety is gradually taking the place of drunkenness, and sickness and mortality are yearly diminishing.

"The establishment of steam communication has perhaps benefited no class of men in a greater degree than the soldier. He can now keep up a frequent communication with his friends, and a tolerable acquaintance, not only with his own native district, but with all that is going on at home, and in European and other countries; and hence we find not a few who are well informed on all the leading topics of the day. Each European regiment of the Honourable Company's service is provided with a well managed savings' bank, a capital school, a well selected library, a printing press, a good coffee shop, and an excellent theatre; whilst those soldiers who are clever and expert workmen as tailors, shoemakers, smiths, farriers, carpenters, bookbinders, watchmakers, etc., meet with every encouragement. There are likewise provided for their amusement chess and cricket clubs, and a fives court built by Government, as well as skittles, quoits, and draught-boards. The men play at 'long-bullets', and are permitted to go out shooting, rowing, and fishing, at their discretion, or as opportunities offer. At this station (Aden) there is, however, no shooting, and little available ground for cricket, or for their favourite pastine 'long-bullets'. With a good library to resort to, with schools where he can qualify himself for promotion and higher employment, and with rational amusements, a soldier's intellectual energies need not now be wasted in dangerous or hurtful inactivity, or his mind left to prey upon itself for want of occupation. Under their beneficial operations, our soldiers are as well behaved in quarters as they are brave before the enemy; and although during the last fifteen months irregularities and drunkenness have been far above the average, yet they are due to a mere temporary ebullition, easily accounted for, and which will gradually subside. Every regiment similarly situated acts in the same way. I am happy, however, to say, that notwithstanding these drawbacks, the deposits in the savings' bank have during the year increased from Rs. 17,700 to Rs. 20,700, whilst the ardent spirits consumed in the canteen have decreased by about two drams per man *per diem*, that is about one-half.

"Improvements in the moral and physical condition of the soldier have progressed as knowledge dawned upon the authorities relative to his requirements. They ever evince the utmost solicitude to add to his comfort, and are unsparing in their endeavours to preserve his health; and, whenever sanitary measures are proposed, they receive the readiest and most mature consideration. In adopting the most efficacious means of preventing disease among the European soldiery, expense is always a secondary consideration....

"Disease is to a considerable extent averted by the strictest attention to his personal economy. He is no longer encouraged, nor permitted, to eat and drink what is noxious; his diet is well regulated, and unwholesome bread and meat are not now imposed upon him by unscrupulous contractors, and passed by officers indifferent to his health and welfare; nor is he allowed to select for his daily rations cheap and unwholesome provisions in the bazaar. Under regulations well considered and rigidly enforced, his ration money is carefully expended; the best articles of diet are provided, and the surplus money returned to him. He is no longer compulsorily initiated into the destructive habit of dram-drinking; nor is he required to pour down his throat a quantity of ardent spirits every morning before breakfast; thus the more surely to induce thirst, and a longing for more spirits, to throw him into a state of fever, and ultimately to destroy the coats of the stomach, and to derange the action of the liver. He can still have two drams daily, beginning after twelve o'clock, but even these he is encouraged to relinquish in favour of coffee, and of sound malt liquor. By a late excellent order, no recruit receives any ration dram until he is dismissed drill. Cleanliness, so necessary for the performance of the function of the skin, is rigidly enforced. Water-sheds are attached to every barrack for ordinary ablutions, and swimming baths are provided in convenient situations, by which the pores of the skin are still more effectually kept open and healthy. The soldier's bedding is an object of daily attention. His clothes are tolerably well adapted to the season of the year, although not of a material suited to the climate, nor of a make admitting of the free use of the limbs and lungs; but, on the contrary, often hurtfully compressing the ribs and abdominal viscera, and affording a very inadequate protection to the person and to the head. The necessity of an ample supply of fresh air to the lungs is now fully understood, and spacious, comfortable, airy, and substantial barracks, well calculated to counteract the effects of heat and climate, have been erected at some, and are in contemplation at other stations of the army; whilst drainage and cleanliness in the vicinity of the barracks are strictly observed. Sanitaria at different stations have been established, and at others experiments to test their utility have, under the authority of the Medical Board, been begun. Some of them have been established with very little regard to eligibility of position, or to any urgent necessity for them; and in some instances the zeal for sanitaria has diverted attention from the requirements of regular cantonments: as, for example, at this station, where there is a capital sanitarium, which may be available for four months in the year for a few sick, whilst the regimental hospital, required every day in the year, is a most miserable and uncomfortable building. The desire is thus, however, equally evinced to benefit the soldier, although I think in a somewhat mistaken direction."

The picture contrasts favourably with the one previously extracted from the pages of the same author; and it is clear to the simplest sense that, if the favourable picture is a correct contrast, the moral, the social, and the sanitary position must be vastly ameliorated and improved, and the British Juggernaut less powerful. Whence, then, the still serious dissolution of men, the loss of an army of 14,000 in the space of eight years in Bengal?

Before this question can be fully discussed, we must, for the sake of those who are not up to medical military statistics, enumerate the modes in which a regiment is destroyed. It must not be supposed that all the men die. The decrement follows in three ways; by *deaths in hospital;* by *deaths out of hospital,* and by *invaliding.* Now, keeping to the loss of the 14,072 in Bengal, for the eight years 1846-54, 5·62 per cent. out of the total force of 156,139 were lost from deaths in hospital; ·74 per cent. from deaths out of hospital; 2·63 per cent. from invaliding. Of the deaths in hospital, 1750 were from fevers; 90 from eruptive fevers; 670 from diseases of the lungs; 68 from diseases of the heart; 816 from diseases of the brain; 3,672 from diseases of the alimentary canal; 1107 from cholera; 74 from dropsies; 19 from diseases of the urinary organs; 86 from venereal affections; 56 from rheumatic affections; 43 from abscesses and ulcers; 136 from wounds and injuries; 11 from diseases of bones and joints; 67 from diseases of nutrition; 1 from disease of the eyes; 18 from diseases of the skin; 2 from diseases of the ear; 40 from drunkenness; and 69 from anomalous diseases; making in all a total of 8,785. Of the deaths out of hospital, 549 were of men killed in action;

9 from wounds received in action; 79 from drowning; 259 from accident; 3 from lightning; 2 from intoxication; 5 from murder; 1 from hanging; 3 from execution; 40 from suicide; 10 from sun-stroke; 70 from apoplexy; 5 from ruptured blood-vessel; 117 in barracks, from causes not specified, 22 from causes unknown (found dead); and 1 from disease of the heart; making in all a total of 1175. The numbers *invalided* amount to 4,112; but it is unfortunate that Mr. Macpherson's tables give the details of the causes of invaliding for four years only, out of the eight, *i. e.* for the four years ending 1853-54. Out, then, of a total of invalided in this period amounting to 2,052 in number, 72 were the results of fevers; 21 of phthisis; 113 of other pulmonic affections; 71 of heart disease; 4 of aneurism; 42 of varix and other diseases of the circulation; 152 of liver disease; 192 of dysentery and diarrhœa; 56 of diseased spleen and other visceral derangements; 34 of hernia; 73 of mental diseases; 43 of paralysis and apoplexy; 40 of epilepsy; 22 of other nervous affections; 5 of dropsies; 29 of diseases of the urinary organs; 48 of venereal affections; 291 of rheumatic affections; 30 of abscesses, ulcers, and fistula; 113 of wounds and injuries; 41 of diseases of bones and joints; 29 of scrofula and scurvy; 182 of diseases of the eyes; 13 of deafness; 55 of expiry of service, or causes unknown; 301 of debility or broken down constitution. Total, 2,052.

Comparing these tables with similar returns from other parts of the world, the average of losses from all the three causes is found to be far above that which occurs in any other of our British troops, whether serving in the colonies or at home.

Entering into a more ample analysis of his facts, Mr. Macpherson explains the influence of age on the mortality of the European soldiers in Bengal; the conclusion being, that below the age of twenty-five the chances of life are better in India and in British America, than in the United Kingdom or the Mediterranean, while after twenty-five they are equal. The period least favourable to life in India, as in England, and in America in a less degree, is that from twenty-five to thirty. The reverse appears to be true on the Mediterranean stations. The influence of length of residence in India on the mortality is also shewn in Mr. Macpherson's tables. His conclusion here is, that of deaths in hospitals, during the eight years already recorded, about one-sixth occurred amongst men who had been less than *one* year in India; more than three-fifths in men who had been less than *five* years; and upwards of four-fifths in those who had been less than *ten* years, Taking it all in all, it may be safely reckoned that fully nine-tenths of the casualties in hospital were in men who had not completed ten years service; and a similar proportion seems to attend the deaths out of hospital.

Of the 2,052 men invalided in the four years 1850-54, 27 were under twenty years of age; 273 from twenty to twenty-five years; 556 from twenty-five to thirty years; 459 from thirty to thirty-five years; 429 from thirty-five to forty years; 192 from forty to forty-five years; 23 from forty-five upwards; and 3 of unknown age. Thus, while nearly three-fourths of the deaths took place in men under thirty, less than one-half were invalided before that age.

As regards length of service in India, of the same 2,052 invalids, 7 were invalided after one year of life in India; 94 after from one to three years; 240 after from three to five years; 242 after from five to seven years; 314 after from seven to ten years; 436 after from ten to fourteen years; 391 after from fourteen to twenty years; 323 after twenty years: and 5 unknown. Thus the period of from ten to fourteen years contributed most to the invalids.

We have already enumerated the forms of disease and accident which give rise to the mortality and invaliding of the European Indian army. Mr. Macpherson, in some very able and judicious comments, enlarges on this topic, and points out the fact, which English theorists would hardly credit, that, so far from rheumatism being a disease almost unknown in India, one-seventh part of the invaliding in the Indian Presidencies is due to that disease.

Up to this point, we have noticed only the casualties of the Bengal European common soldier. A word about the officers must now have place. Mr. Macpherson has here another instructive table for our guidance, the pith of which is, that the average strength of the officers, for the eight years 1846-54, was in round numbers 712, and the average deaths fifteen, or 2·11 per cent.

Certain other points connected with Mr. Macpherson's statistics, of less importance, perhaps, than those we have supplied, but still most interesting, offer themselves for notice. We must not enlarge here; but, at the risk of repeating some things that have been stated, and to avoid the danger of having omitted the essence of our author's remarks, we give verbatim his own *resumé*. It runs as follows:—

"1. That European troops, serving in the Bengal Presidency, lose annually, from one cause or other, 9 per cent. of their number: viz., about 5·62 by deaths in hospitals, 0·74 by deaths out of hospitals, and 2·63 by invaliding.

"2. That, of the men constituting the European force, two-fifths are under 25 years of age, more than one-third between 25 and 30, and nearly one-fourth upwards of 30: the proportion of men in the middle period being much larger in India than elsewhere.

"3. That below the age of 25, the chances of life for soldiers are better in India and in British America than in the United Kingdom or the Mediterranean; while, after 25, they are nearly equal.

"4. That the period least favourable to life in India amongst soldiers, is,—as in England and America likewise in a less degree,—that from 25 to 30. This is not true of the Mediterranean stations, where the ratio of deaths according to age at all periods is more equable than elsewhere.

"5. That, of the soldiers who die, about one-sixth have resided less than one year in India, more than three-fifths less than five years, and upwards of four-fifths, in fact, fully nine-tenths, have not completed ten years in this country.

"6. That the proportion of deaths out of hospital, even excluding the numbers of those 'killed in action' or 'died of their wounds', etc., is larger in Bengal than in the Colonies.

"7. That the average annual number of suicides among soldiers is about five,—rather a smaller proportion than in most other countries.

"8. That the average annual number of men invalided in Bengal is about 2·63 per cent. of all strength, or 0·68 higher than in the Mauritius, and more than one per cent. higher than in British America, but less than in the cavalry and household troops in England. Of the latter, however, a large number take their discharge on the expiry of their term of service, without any medical reason.

"9. That the principal causes of invaliding in this presidency, are, general breaking down of the constitution and rheumatic affections, each of which yield about fourteen and a half per cent of the whole; the next in order being bowel complaints, eye diseases, liver affections, diseases of the respiratory organs, wounds and injuries, and mental diseases.

"10. That, as might be expected, bowel complaints and liver affections combined, yield one-sixth of the whole number invalided, being more than double the amount at the Cape, nearly eight times the number in the Mediterranean, and more than ten times the number in British America.

"11. That, on the other hand, diseases of the respiratory organs give two and a half times fewer men to the invalids than the same class at the Cape, and five times less than in British America and the Mediterranean.

"12. That one-fifth of the men invalided are under 25 years of age, more than one-fourth between 25 and 30, and more than one-fifth between 30 and 35, and a like number between 35 and 40. So that less than half of the men invalided are under 30, whereas nearly three-fourths of the deaths take place under that age.

"13. That, of soldiers invalided, sixteen and a half per cent. have served under five years, forty-three and a half under ten, and eighty-four per cent. under twenty years: the period of from 10-14 contributing most, and that from 14-20 the next largest proportion.

"14. That, (*a*) the average proportion of sick to strength is, in soldiers, 204·36 per cent.; in officers, 132·25; in women, 126·81; and in children, 78·81.

"(*b*). The average percentage of deaths to strength is, in soldiers, 5·62; in officers, 2·11; in women, 4·45; and in children, 8·41.

"(*c*). The average ratio of deaths to treated is, in soldiers, 2·75; in officers, 1·60; in women, 3·51; and in children, 10·68. While, therefore, the amount of sickness is nearly three times as great in soldiers as in children, its intensity is four times as great in the latter as in the former.

"15. That, both in soldiers and officers, by far the largest amount of sickness is caused by the same classes of maladies: viz., fevers, diseases of the alimentary canal, venereal affections, and wounds and injuries.

"16. That fevers cause the largest number of admissions to hospitals in women and children, as well as in soldiers and officers, giving fifty and a half per cent. in women, and forty-one per cent. in children.

"17. That the classes which contribute most to the general mortality are the same in soldiers, officers, women, and children: viz., diseases of the alimentaary canal and fevers.

"18. That the classes which prove most mortal to those attacked are, in soldiers, cholera and eruptive fevers; in officers, cholera and dropsies; in women, likewise, dropsies and cholera; and in children, diseases of the brain, etc., and cholera.

"19. That, as had been remarked by M. Boudin in regard to tropical countries generally, the annual number of admissions to hospitals, and of deaths, in Bengal, range within very wide limits from one season to another."

The reader is thus in fair possession of the condition of European soldiers in the Bengal Presidency, up to a late date. From all we can gather, the position of the soldiers elsewhere in that vast empire is much of a muchness. Having, therefore, the leading facts regarding the decrement of Indian troops, what facts are known about the causes of such decrement? On this great question, Mr. Macpherson makes but two statements of moment; but these, brief as they are, must be accurately noted.

1. Comparing the losses sustained by the army in the colonies and at home with those in India, he tells us that "climate alone, if the troops were in all respects on equal footing, would not produce the great disparity observable in the rates of mortality. What swells the table of losses is, the great amount of duty in the field which men serving in India have to undergo, compared with their brethren in arms in the colonies; and the extraordinary risks of sickness incurred by them, in occupying new positions before there has been time to provide fitting shelter."

2. In his *resumé*, when stating the fact that the average percentage of deaths to strength is amongst the soldiers as 5·62, to 2·11 amongst the officers, he adds in a foot note a rather telling part of the text, that the small mortality among the officers is due to the greater facilities they enjoy of obtaining change of climate when they fall sick.

Leaving Mr. Macpherson with these his cautious hints, we turn again to Dr. Arnott for his enumeration of disease causes. It is fortunate that Dr. Arnott's observations extend over the same period of time as Mr. Macpherson's statistics. Dr. Arnott's information, however, is derived from one regiment; but as this regiment, in all its vicissitudes and perambulations, affords a fair type of other regiments in India, our comparisons from it are again an approximation to the general rule. We will introduce the regiment in the words of its historian.

"Since 1846 the Bombay Fusilier regiment has been in no wise favoured with respect to climate, locality, accommodation, or duty; but, on the contrary, it has been exposed to all the ordinary causes of sickness and loss in no minor degree, including the casualties of war, and the ravages of epidemic disease. It has served within and without the Honourable Company's territories; within and beyond the tropics; and in climes ranging from 13° to 34° of north latitude, and from 44° to 74° of east longitude. It has been stationed on the shores of Arabia, and in Scinde, and in far inland provinces. At places where, for the greater portion of the year, the cool and moist sea breezes prevail; again, at stations on the highlands of the Deccan, 1,800 feet above the level of the sea, where for a similar portion of the year the arid hot winds, surcharged with dust, sweep over its vast bare plains, and where the skin, nails, and nostrils are parched, curled, and dried up. The fusiliers have taken their share in the field of battle, and in the performance of ordinary garrison duty; they have made long marches, and have been again stationary; they have enjoyed a profusion of supplies, and have been limited to commissariat rations. The regiment has had its ranks thinned by the fire of the enemy; it has been exposed to great vicissitudes of temperature, in crowded tents with the thermometer at 114°, and a few months afterwards at freezing point, with snow all around. It has been conveyed from place to place, sometimes in small river steamers, and sometimes in large sea-going vessels. It has been at times located in small, confined, and comfortless pendals, and at others in lofty, capacious, and commodious barracks; and again in pendals which have been described 'a disgrace to the Government of India.' I may add that I have seen sickness prevailing so little, that for two months the admissions into hospital did not reach 12 per cent. of the strength; and, on the other hand, I have seen, in the same space of time, the admissions into hospital exceed the entire strength of the regiment, at the rate of 120 per cent. With a strength exceeding a thousand men, I have known six consecutive months pass over with only one burial; again, I have witnessed funeral parties consign to their last resting-place forty-eight of their comrades, lost by disease in two consecutive days. I have seen the regiment decimated by disease in nine days, and reduced in the space of a year, from all causes, at the rate of 215 per 1,000, which would have annihilated it in less than five years; and I have seen the annual reduction so low as 47 per 1000,—a rate of decrement which would require twenty years for its absorption. I have known the 'discharged' from the service not exceed one in twelve months, and by the operation of the late order I have seen them amount to twenty-two. But most of these points will be adverted to hereafter in detail. Enough, I think, has been said to show that since 1846 the Fusilier Regiment has escaped few of those influences which are deemed pernicious to the health of the European soldier in a tropical climate; so that, irrespective of the importance of the specific question under consideration, the history of the regiment for that period may be deemed interesting, as exhibiting the changes to which a large body of European troops, performing the ordinary duties required of soldiers in India, and exposed to the various sources of sickness and mortality incident to men thus actively engaged, are liable."

In the course of their changes, during the period of time thus marked out, the regiment underwent the entire metamorphosis of the Irishman's knife. On the 1st of April, 1846, the strength of the regiment, officers excluded, was 791. In the eight years it was joined by 845 new men; and, in the same time, it lost 796, or five more than the original number. Of the lost men, 365 were from deaths, of which only 23 were from battle wounds; 87 were invalided; and 29 were invalided and pensioned. Amongst those invalided, mania and rheumatism hold a high figure as the disposing cause. Forming a general estimate, Dr. Arnott shows that the entire loss of his regiment from all causes was 796, out of an average strength of 957, which is at the rate of about 104 per thousand per annum, and "*equal to the entire absorption of the regiment in nine years and seventh months.*" Some deduction from transfers require to be made in the case of the Company's regiment; but the result is little less startling, when all deductions are included. Three score years and ten may be taken as the life of a man in the world at large; truly so. But your European Indian soldier gives this exception to the rule, that the three score has to be left out when he is mentioned. Such is the extent of human sacrifice to our great Juggernaut.

Now to the reasons of this decrement. We may exclude actual fighting almost entirely, because the numbers lost in a sharp campaign would all be losses superadded to those we have named, or nearly so. What, then, are the reasons? They are included mainly in one sentence—neglect, sheer neglect of sanitary law.

We saw Dr. Arnott, a short time ago, describing in glowing and general terms the favourable contrast which obtains in these days in the sanitary care of the troops, as compared with the days when India was the dead-house of all European soldiers who visited it. Not for the sake of convicting Dr. Arnott of misrepresentation or contradiction (for we know that he is guilty of neither), but for the sake of following an author wedded to the service; and for indicating how wide a breach may occur between the general and the particular, we shall follow him exclusively in showing the true sanitary condition of the European soldier in India in modern times. Every syllable we shall give will be but the condensed and accurate statement of this learned officer.

*Barrack accommodation* is of vital importance in India; but it is only in one or two of the newest stations that there is such a thing as good barracks. In some cases, ventilation of barracks is carried to such a pitch of refinement, that it is assisted by gutters opening under the wall outside, and near the centre of the floor inside. These gutters are the fitting receptacles of dead rats, and such-like sanitary improvements; and the men at times are overpowered by the putrid frankincense to the mighty firm of cross-bones and skull. Their walls and roofs prevent equalisation of heat, and are specially mischievous in tropical lands; but at Aden, from this cause, no soldier can sleep in his barrack in the hot weather; so, as the sun goes down, he spreads his bed in the open air, and is exposed to the damp—a great, but lesser, evil. In these hovels, during hot weather, the thermometer has stood at 103°; whilst in a common native building, with good walls and roof, it never rose above 95°

In the *out-buildings* connected with Indian barracks cleanliness and drainage are but little cared for. There are many barracks furnished with only one small room in the corner of a narrow verandah; and here are stowed away water casks, in which men are expected to perform their ablutions: "and, incredible as it may appear", says Dr. Arnott, "I have seen iron vessels to receive urine, standing side by side with casks holding the water the men were to drink. Fœtid ammoniacal vapours abounded, which strongly impregnated the drinking water, and formed a reasonable cause of complaint."

In communion with Indian barracks are certain little Edens, called *patchonis*. These are for the lodgement of the married soldier. They are placed on the flank of a range of barracks, and are too often most mean and uncomfortable. To this fact, the high rates of mortality which everywhere prevail amongst the women and children of a barrack are greatly attributable. In some instances, these huts are so ill built, that they evaporate under a monsoon; others are so confined, that a man with children has barely space to move about or lie down. They are so low, that, to heighten them, the floors have to be dug out. The rain which falls from the eaves soaks in on wet days so thoroughly that a mud carpet is the result; there is water everywhere, and in the hot, damp air, the flaccid body of the occupant is lightly steamed. The dry, hot day comes, and the little place is converted into a mild oven, in which the said body is mildly baked. The ladies who are subjected to these enlivening processes, die at the rate of 6 per cent. per annum. The children are removed at the rate of 7 per cent.

*There are cells* in every European cantonment in India, where the wicked are punished. These cells are never improved, but they are very often occupied, specially by men exhausted by debauchery and the depressing passions. The cell is a hole above ground, with the stereotyped iron gratings and doors, but no verandah; so that when the sun looks in upon the culprit to see how his crimes are disappearing, the place is insufferable, so unutterably fiery. Prisoners and culprits get no sympathy, and measures to ameliorate their miseries are repudiated. This, argues our excellent historian, can scarcely be correct; "for no punishment is supposed to have an injurious effect on a man's health." What does Dr. Arnott say to hanging, then? On one occasion it was necessary to keep a mad-cell prisoner, who was after committing suicide, under the eye of a sentry. The sentry, worse off than the prisoner himself, stood exposed to the direct rays of the sun by day, and to the damp and cold at night.

There are *baths* in Indian cantonments which are so badly constructed, and so small, that, as one infers, a regiment could not march into them in less than a month, although the men went in turns and in the most perfect order. One of these baths, which cost a great deal of money, was so repulsive, that it was only likely to be sought out by some man in the fever of suicide, who would end himself quietly, and without the appearance of anything more than an accident in ablution.

On *shipboard*, the condition of the Indian soldier is a novelty in its derangements. The crowding and absolute misery of the men exceed belief. In the voyage of the Fusiliers to Aden, the only place in the steamers where the men could spread their bedding was on the top of boxes and gun carriages. They had to occupy the middle part of the vessel, where a constant stream of dirty water or spray and high waves were washing over the decks, and made it impossible there to sleep. Some men had thus not a night's rest on the voyage.

Let the shipboard pass; it is an incident not often repeated, comparatively—we come to a thing of every day life—the dress. Not an attempt here, Dr. Arnott, at condensation; you shall have the inverted commas, and tell the British public (which would be the better for a closer acquaintance with you by the way) your story in full:—

"*Dress.* Next to barrack accommodation, no subject is deserving of more consideration than the dress of the European soldier in India. I have said that it has undergone gradual improvement, and such is generally admitted to be the case; nevertheless, much remains to be done. Improvement is slow, and indeed the pertinacity with which people adhere to old habits is nowhere more remarkable than in the manner of dressing the European soldier in the torrid zone. The soldier in India is belted and clothed like the soldier in Europe, and consequently in a manner calculated to make duty irksome to him, and to be prejudicial to his health and comfort. He must still dress in the cloth coat of a northern climate, in trousers fit for a Siberian campaign, and wear a hat that protects the head from neither heat nor cold. Efficiency is sacrificed to appearance, and soldiers serving in latitudes having a mean annual temperature of 50° above zero, and in countries with a mean annual temperature of 50° above freezing-point (that is of more than double the number of degrees of Fahrenheit above freezing-point) are dressed alike. Surely this is taxing the wonderful capacity of 'man adapting himself to all climates' to too great an extent. Sir C. Napier wished the soldier to be always ready to go into action, and to appear on parade in fighting trim; but he did not consider 'full dress' prepared the soldier best for either. . . . . With the most striking unanimity, all who are able to consult their own feelings adopt a costume calculated as much as possible to moderate the effects of increased temperature; and custom has introduced, and authority has sanctioned, one of comparative comfort. . . . . Common sense, the commonest feelings, nature herself, all point out the necessity of the alteration, whilst dearly bought experience has amply proved that it cannot be neglected with impunity. If, then, it is found after long experience that such a costume is requisite for people pursuing avocations requiring little personal exertions, and which seldom lead to exposure in the open air, how much more necessary must a similar description of dress be for men loaded with a musket and heavy accoutrements, under a mid-day tropical sun. In the face of experience, however, and opposed to common sense, with the most strange and unaccountable obstinacy, the poor soldier, who has no option, is still doomed in India to be clothed as if he were in Europe. Every one will admit that to preserve the ideas, the views, the associations, the pride, and the appearance of the British soldier must be a paramount object; but that any of these would be lost by adopting a style of dress better suited to this climate may fairly be questioned. . . . The present 'full dress' is not adapted for this country, and the sooner it is altered, or entirely abolished, the better. The stiff hard stock has happily been done away with; and the cumbrous chaco, with its heavy metal ornaments, has been at home, as in this country, almost as universally condemned, so that it is hoped it will soon share the fate of the stock. Lord Hardinge, in his evidence before the Parliamentary Committee on Indian affairs, related that the men of H.M.'s 9th regiment, in the second Affghan campaign, 'marched and fought in their forage caps, through the Khybur Pass to Cabool, and back again from Cabool to India, having carried their European chacos on camels the whole distance up to Cabool and back again at great expense and inconvenience'; and his lordship seemed to adjudge great credit to the gallant regiment for having done so. The zeal and devotion thus evinced were most creditable; but it appears not a little extraordinary that this gallant corps should have been put to such a shift. When the 1st Bombay Fusiliers went on field service in 1848, all the chacos and full dress coats were left behind, and every man marched and fought at Mooltan, and through the Punjaub to Peshawur, and back again to India, in forage caps and shell jackets. Each one would have heartily rejoiced had he been permitted to continue a dress which becomes a well shaped soldier, and in which he knew by experience that he could properly perform every species of duty. But such an indulgence could not be allowed; and the rats, in his absence, having damaged or destroyed the old full dresses and chacos, each man had to reprovide, and incommode himself with these expensive and useless articles. If marching and fighting without chacos be an improvement, as it has been acknowledged, why is such a head-dress not entirely discarded? But even so late as the last Burmese campaign, some European regiments (if we may trust the accounts in the papers) went into action in full dress, and in consequence of such infatuation, these corps lost also as many lives by *coup-de-soleil* as by the fire of the enemy. The helmet which has been proposed—if anything besides the forage cap with white cover is required—will be a great improvement. The red frock coat, which is spoken of at home as a substitute for the present full dress coatee, will also be a great improvement, and if made loose, will allow of the free use of the muscles of the upper extremities, and a free expansion of the chest, which the present coatee does not. It will also, no doubt, be denuded of many useless ornaments, which add so much to the weight, as well as of the fantastic shoulder appendages, which increase the inconvenience of the present one, and so effectually prevent the soldier, whilst incased in it, from lying down, or attempting to take rest in the recumbent position. That the new pattern, with all its improvements, will answer in this country, seems problematical. The quantity of material and its weight (if made of such cloth as is now in use) will be insuperable objections. Why the cloth trousers are retained as part of the soldier's dress is inexplicable. No soldier can march in them, they are not worn on parade, and indeed they are not worn above six times a year at all, and could well be dispensed with. A cloth of a much cooler and lighter description, such as serge, could easily be procured for use in India both for coats and trousers, and should be substituted."

It is cheering to find, after the account of the dismal facts about barracks, patchonis and dress, that, at all events, the *rations* of the Indian army are good, and that in this respect the recommendations of the medical officers have been so far attended to, that no physiologist could reasonably complain of the diet roll in so far as it relates to *matter*. In respect to manner, however, his complaints might be grievous, especially did the mysteries of Epicurus form part of his studies; for the cooking rooms in barracks are so atrociously constructed, that smoke is always in the ascendant, and eyes are always in conjunction. There is no escape for the smoke, so it blinds the cook, who thereupon spoils the good rations in innocent obscurity. The hospital diet is good. The *water supply* varies; generally it is unlimited and good. At Aden, Dr. Arnott found the men in his regiment allowed a gallon of fresh water and two gallons of brackish water per day, with lime juice and pickles as an antidote, so that the bad water might not give the scurvy. Ingenious certainly. Our author thought this plan too round about, such is the perversion of intellect, and recommended the removal of the pickles and the supply of plenty of pure water. The recommendation was attended to; and the result was, that only two cases of scurvy occurred, and both in men, volunteers, who had suffered from the disease at Aden. Great advantages have accrued to the soldier from the substitution, in some parts, of sound malt liquor for ardent spirits. This substitution has led to a diminution of crime, sickness, and mortality.

In the matter of *occupation* and *amusements* for the soldier in India, especially when he is not out fighting, much remains to be done. The man "has still too much time on his hands, and idleness is the bane of his existence." Tradesmen should be allowed to practise their avocations; and those who know no trade should be taught one. Healthy games should be instituted, and gardens cultivated. All these and other things require to be done, before the idle Indian soldier is kept *in* health and *out* of mischief.

The invaliding process in the Company's service is conducted on a worse principle than that which obtains in the Royal service. There is no increase of pension for good conduct; and when men are deemed unfit through sickness for active service, they are for ever removed from the strength of the regiment. Many soldiers thus discharged would be glad to return to India, if their health became re-established by a year or two of absence. As it is, they are lost. Still more important in this respect is the question of *furloughs.* European officers and native troops, beautiful sepoys, to whom in these days we are so much indebted, are provided for liberally on this score. But the European soldier has no furlough. There he lives on, in hopeless exile, until invalided or discharged. The act is brutal —there is no other word that expresses it. Dr. Arnott suggests that every soldier, who possesses a certain number of good conduct rings, should be allowed a two years furlough to Europe after ten years service. No other measure would tend so much to reconcile soldiers to their duties, ensure their good conduct, prevent that longing for home which produces, directly and indirectly, such a numerous class of dyspeptic and other complaints, and exalt the character of the service. Whilst at home, they might, from their former good conduct, be very usefully employed in the various military establishments of the Honourable Company, as non-commissioned officers, drill instructors, and the like; indeed, it is strange that the various staff appointments in the home establishment are not conferred upon meritorious Indian soldiers, instead of favourites of no actual experience or merit.

Dr. Arnott, in following out his history of the Fusiliers' adventures, gives a deplorable picture of the position of the troops stationed at Aden in 1854. The place naturally had many advantages; but the barracks and other arrangements at that time were unfit for beasts of the jungle. In a triangular space, bounded by the sea on the east, a nullah on the south, and having a high hill as the base, each side not exceeding four hundred paces in length, were situated pendals for not less than 500 men, as well as the married men's houses, the hospital, the officers' mess room, and some of the bungalows. The position was low and hot. The barracks were of mats, cut so small that there was not space for tables for the men to have their meals at. On two occasions, when the rain came, a stream broke through one barrack; pools of water stood in others; and next morning, in one of the guard-rooms, the men's cots were floating about in several feet of water. To this low and hot position, and to the faulty condition of the barracks, our author attributes several cases of sun-stroke, which occurred annually, and from which the artillery, which was better placed, had not lost a man.

Having thus sketched out, from Dr. Arnott and Mr. Macpherson's reports, the serious sanitary deficiencies by which the decimation of Indian armies is mainly ensured, we might leave them here, were there not one other fact, which we would gladly omit, but which must in honesty be told.

This last fact is, that venereal affections are among the leading causes of sickness in the European Indian troops. Mr. Macpherson says that, amongst both soldiers and officers, by far the largest amount of sickness arises from four causes: fevers, alimentary diseases, venereal diseases, and wounds and injuries; but, in another place, he shows that of venereal affections there are about 1 per cent. less in the officers than in the men. Dr. Arnott, entering more fully into this question, thus writes :—

"There still remains a class of diseases on which a few observations may be offered. Although only one death during the period under review is placed under diseases of the sexual organs, yet the extent to which the efficiency of a regiment is affected, the number of men constantly attacked, the deterioration of their constitutions, and the consequent increased liability to future disease (as well by the nature of the affection itself, as by the long confinement in a sick ward), the losses to the State by men dying, by their being invalided on account of these complaints, as well as the expense incurred in the medical treatment, give to syphilitic diseases a very important place in the medical economy of a European regiment."

And then, having given a table of cases of this disease, he continues :—

"Irrespective, then, of the deterioration of the constitutions of fifteen men daily, from venereal affections, and of the diseases to which they indirectly give rise, it appears from the return given in a previous part of this report, that upwards of $1\frac{1}{2}$ men are annually invalided on this account, and likewise for the last return that one in the regimental hospital, and three absent from head quarters, died of them. The State has therefore in that period incurred a direct loss of rs. 5,100, supposing each man to be worth rs. 300, or 25 per cent. less than the highest price required to purchase a soldier's discharge from the service. To this must be reckoned the loss of the services of fifteen men daily, the expenses whilst in hospital, and the charges of sending many away for change of air. When these items are added together, I have no doubt that the cost so incurred if properly and judiciously laid out, would not only prevent much bodily suffering, but prove a great pecuniary saving to the State."

Finally, Dr. Arnott points out that the cause of this great evil, irrespective of the moral question, lies in the fact that the Government discourages marriages, and allows, indeed, but 12 per cent. of married men in each regiment; the which, as we have seen, it treats worse than the wildest of its unmarried scapegraces.

Reviewing the historical facts copied above, a host of grave and serious thoughts rise up in sad and dismal array. Does anyone wish to hear the other side, we will be candid, and will at once refer such one to the handsome sergeant who basks under the court in the busiest street in London, inviting listlessly all young men who near him to be daguerreotyped into the likeness of the glorious dragoon, whose noble presence is depicted on the screen which bedecks the court entrance. Said sergeant being up to his work, will meet us on even terms, and will paint in Eastern symbol the Indian paradise. Good: but till the sergeant is heard, let us speak another word about Juggernaut Britannicus.

It is clear that, irrespective of actual loss in fighting, or nearly so, a European Indian army of 14,000 men is lost to a man in eight or ten years. It is clear, too, that the influences which give rise to this loss, are mainly sanitary, and therefore remediable. But the climate—ah! there's the rub. Well, the climate is bad certainly, not because it is the climate fighting hand to hand with the man, but because the climate exaggerates disease causes, and makes sanitary measures doubly necessary. Are our statesmen asleep, that they look not to their true enemies in the East? We hear of sepoys in arms, and of forces sent out to meet sepoys with rifles and cannon and all the paraphernalia of war. But the choleras and the fevers which are in open mutinies with us, what of them, or of the forces sent out against them? Not a word, save that the men, armed to the teeth against the sepoy, are going against the sun in armour fitted for the pole. But let us compute losses by common sense rule, and see how these sanitary details, which weigh so little in government scales, affect the position of our affairs in India when taken in their vastness. Let us suppose for the moment that at this time and for the year to come not a sepoy should give trouble. Let us say, by an approximate but theoretical figure, that on this day, October 1st, there are sixty thousand European soldiers on the surface of India. Let us, lastly, calculate by the eight years statistics supplied by Arnott and Macpherson, and we shall find as the result, that at the least, from six to seven thousand men of that army will be inevitably lost from natural causes by October 1st, 1858.

Our friend the sergeant, with the winning smile and the paradise tongue, does not think this loss very considerable, he tells us, all things considered. Perhaps not. But think of India in general mutiny. Think of the sixty thousand under the uncovered sun; think of short commons, and hard muscular fighting; of broken rest and irregular life; and what then? Think of hospital epidemics from pyæmia and erysipelas amongst the wounded, apart from epidemics common; and is not the inference clear that the disease-causes will double, treble, quadruple in their intensities? In a word, should this present mutiny continue till October 1858, which God forbid, the estimated losses from disease may rather be taken at thirty thousand to every sixty thousand, than six, to which will have to be added the losses on the field, when battle after battle, and sortie after sortie, and siege after siege, shall have closed.

We must stop. The subject of argument is such as none can ignore. India, as a British possession, rests now primarily on the sanitary law; secondarily, on the sword. What is the sword without the healthy arm? In a war of years against the natives of India, how long can England send out her two men for one; the one man for her own pestilence Juggernaut, the other man for the chastisement of sepoy scoundrels? This is the great problem; and the sooner the pestilent element of it is considered, the better for us all at home and abroad.

Meanwhile, a final word to the vengeance-men and the sword-proselytes. To the first, this word: When the vengeance fit is on, and the avenging fiend is filling your souls to madness with the idea of rebels slaughtered by the hecatomb; temper the rage, by reflecting how much more noble were an effort, however weak, to rescue your own thousands of brave ones from avoidable death. And to the second, this: When you preach, in the zeal of your house, Down, down with the Indian Juggernaut! preach also, Down, down with the British Juggernaut in India; and expound boldly the nature-written fact, that, in the physical, equally as in the moral world, the laws of the Eternal can never be broken with impunity.

